# SERRS multiplexing with multivalent nanostructures for the identification and enumeration of epithelial and mesenchymal cells

**DOI:** 10.1038/s41598-020-72911-w

**Published:** 2020-09-25

**Authors:** Lucio Litti, Andrea Colusso, Marcella Pinto, Erlis Ruli, Alessia Scarsi, Laura Ventura, Giuseppe Toffoli, Marco Colombatti, Giulio Fracasso, Moreno Meneghetti

**Affiliations:** 1 Department of Chemical Science, University of Padova, via Marzolo 1, 35131 Padua, Italy; 2 Department of Medicine, University of Verona, P.le L.A. Scuro, 37134 Verona, Italy; 3 Department of Statistical Sciences, University of Padova, via Battisti 241, 35121 Padua, Italy; 4 SOC Farmacologia Sperimentale e Clinica, Centro di Riferimento Oncologico, Via Franco Gallini 2, 33081 Aviano, Italy

**Keywords:** Diagnostic markers, Nanostructures

## Abstract

Liquid biopsy represents a new frontier of cancer diagnosis and prognosis, which allows the isolation of tumor cells released in the blood stream. The extremely low abundance of these cells needs appropriate methodologies for their identification and enumeration. Herein we present a new protocol based on surface enhanced resonance Raman scattering (SERRS) gold multivalent nanostructures to identify and enumerate tumor cells with epithelial and mesenchimal markers. The validation of the protocol is obtained with spiked samples of peripheral blood mononuclear cells (PBMC). Gold nanostructures are functionalized with SERRS labels and with antibodies to link the tumor cells. Three types of such nanosystems were simultaneously used and the protocol allows obtaining the identification of all individual tumor cells with the help of a Random Forest ensemble learning method.

## Introduction

The identification of circulating biomarkers in different body fluids is an important step toward non invasive diagnosis for monitoring the dynamical evolution of tumors and for their treatment^1^. The metastatic spread of tumor cells from the primary mass is considered a leading cause of cancer-related death^2^. These cells represent a heterogeneous population of cells that are able to survive in the bloodstream and to initiate metastatic growth of new tumors^3–5^. Some of them have been validated as prognostic markers for metastasis of different types of tumors^6^. Recent experiments also suggest the importance of the presence of cell with mesenchymal markers, and not only with epithelial markers, in the blood^7–9^.

The capture and analysis of tumor cells in the blood are challenging tasks because of their low abundance^10^ and relevant obstacles are found today for technologies to be used in clinical diagnostics^11–15^.

Nanotechnologies can help to face the challenges for the detection and analysis of these type of cells^16^. Assays based on highly specific and sensitive surface-enhanced Raman spectroscopy (SERS) technology are very efficient to detect these cells^17–20^. One of the crucial advantage of SERS over other techniques (usually fluorescence-based assays) is its sharp fingerprint-like spectral pattern, which are clearly different from interfering signals of the complex biological environment^19,21^. The narrow SERS signals^22–28^ make possible a simple multiplexing approach with only one exciting wavelength in resonance with the localized plasmon resonance of the nanostructured system. This allows obtaining the SERS signals of molecules used as SERS reporters, which strongly interact with the plasmonic system^29–31^. Moreover, SERS signals do not suffer from bleaching, which is usually present in laser induced fluorescence. SERS signal intensities can be further increased, obtaining the surface-enhanced resonance Raman scattering (SERRS), when the laser is in resonance both with the localized surface plasmon resonance of the plasmonic nanostructures, and with the reporter molecules^31–33^. It is worth mentioning that the presence of a plasmonic structure usually suppresses fluorescence signals of molecules close to the nanoparticles (NPs), making background signals negligible. We have already shown that the opportunities given by the SERRS approach can be successfully exploited for specifically identifying prostatic tumor cells expressing prostate specific membrane antigen (PSMA) and prostate stem cell antigen (PSCA)^26,31^ or other antigens using engineered peptides^25,34,35^. The functionalization of plasmonic nanostructures with a large number of antibodies (Abs) increases the targeting activity with respect to isolated Abs^36,37^. This interesting possibility can be exploited also using different types of Abs on the same nanostructure, which allows to further increase its avidity^38^.

SERS signals for identification and enumeration of tumor cells were exploited by some laboratories without^17,23,39^ or following an enrichment step^40–44^. Multiplexing approaches^45^, coupling SERS and fluorescence^46^ were also considered for cells identification^47^.

In the present work, we address the possibility of identifying and enumerating tumor cells with epithelial and/or mesenchymal markers, using SERRS multivalent nanostructures and the Random Forest ensemble learning method. Abs recognizing EpCAM and E-Cadherin (E-Cad) were used on the same nanostructure for epithelial cells identification, whereas CD44 and N-Cadherin (N-Cad) were used for the mesenchymal ones^48^. The choice of using more than one Ab on a single nanostructure allowed enhancing the avidity of the nanostructure for a cell phenotype and to simplify usual analysis in which a single Ab was used. A third type of nanostructure was used to identify PBMCs, with an Ab recognizing CD45, an antigen broadly expressed on white blood cells. The third type of nanostructures was introduced because it was not possible to completely deplete PBMCs from the spiked samples used for the experiments. Each type of nanostructure was functionalized with a different SERRS reporter molecule to permit their identification in a multiplexing approach. SERRS signals recorded for individual cells were analyzed with a Random Forest ensemble learning method^49^, which allowed identifying the tumor cell phenotypes, also giving the level of identification.

All the needed functionalizations of the nanostructures were greatly simplified by using gold nanoparticles synthesized by laser ablation in solution, which do not need surfactants or other stabilizing molecules^50–52^. The naked surfaces of the nanoparticles simplify their full functionalization because it does not require control over the ligand exchange. Furthermore, the naked surfaces favor intense SERRS signals because of the strong interaction with the reporter molecules.

To produce spiked samples, we used LNCaP prostatic tumor cells, as model for the epithelial phenotype and U251 cells as model for the mesenchymal phenotype.

The present approach allows further analyses on the tumor cells after their identification, because they are always individually available on a glass slide. This is also an important step, which is not possible with other approaches like those based, for example, on PCR protocols.

## Results and discussion

### Nanosystem synthesis

**Figure 1:**
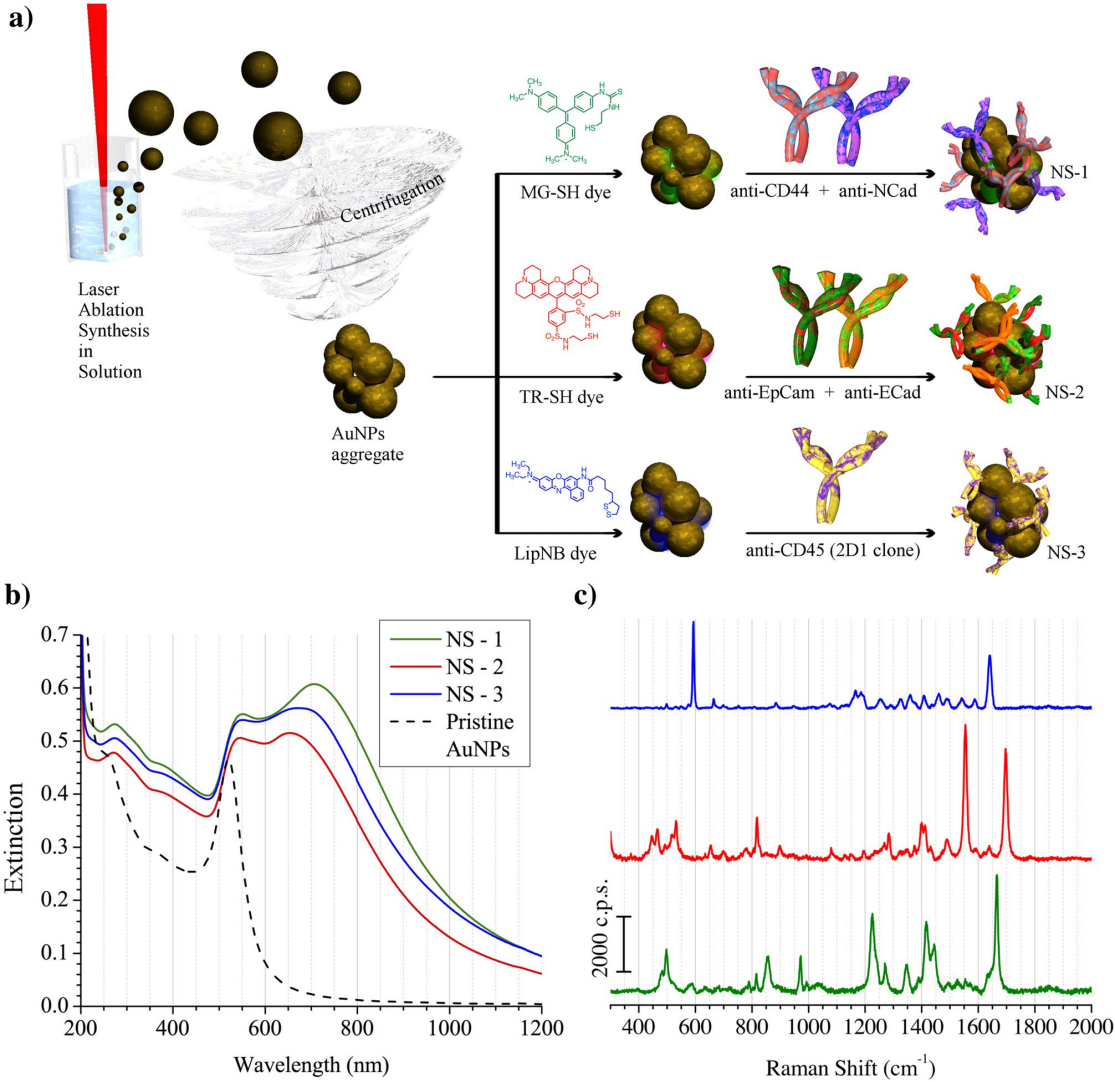
(**a**) Schematic representation of the synthesis of NS-1, NS-2 and NS-3: LASiS, aggregation by centrifugation, functionalization with a SERRS reporter molecules and then with the Abs. (**b**) UV–Vis-NIR extinction spectra of the three nanostructures, showing the characteristic plasmon resonances of aggregated NPs in the NIR. The extinction spectrum of the not aggregated NP, as obtained by the LASiS synthesis, is reported with a dashed line. (**c**) SERS spectra of the same nanostructures (NS-1, NS-2 and NS-3 from below) in quartz cuvette exciting at 632.8 nm.

A schematic representation of the synthesis of the nanostructures used in this work is reported in Fig. 1a. Gold nanoparticles (AuNPs) were obtained by the laser ablation synthesis in solution (LASiS). This synthesis is performed directly in water and the nanoparticles do not need to be stabilized with molecules, like citrate or thiols, because they are produced with native surface charges which, by coulomb interaction, stabilize the colloidal solution^50^. We obtained AuNPs with nanomolar concentrations in water by focusing 9 ns pulses of a Nd:YAG pulsed laser at 1064 nm on the surface of a pure gold plate under water. It was already shown that AuNPs have dimensions of about 25 nm and a ζ-potential of about − 30 mV^31^. According to a previous defined protocol^31^, a centrifugation of the colloidal solution allowed to obtain a controlled aggregation of the AuNPs with clusters containing, on average, 10–20 AuNPs and dimensions of the order of 100–200 nm (see Supplementary Fig. S1). The plasmonic properties and the strong SERS enhancement of the aggregated AuNPs were already analyzed in a recent work^53^. The aggregated AuNPs can be easily re-dispersed with a sonicating bath. A tiny amount of a thiolated dye was linked to the AuNPs to label them with a SERS signal. From the dye library, that we already considered in previous works^31,54^, Malachite Green, Texas Red and Nile Blue were chosen in the present case as reporter molecules for the three type of nanostructures (see Fig. 1a). Since these dyes show absorptions close to or in resonance with the excitation laser at 632.8 nm, used for the SERRS measurements, the nanostructures showed high intensity signals.

The SERRS nanostructures were then functionalized with antibodies (Abs) as targeting agents. To obtain a better link with the gold nanostructures, Abs were first functionalized with 2-iminothiolane to introduce, on average, about 1–2 new thiols per antibody^31^. This procedure was found effective to preserve the affinity and specificity of the antibodies and to obtain a total binding of about 300 Abs/SERRS nanostructure.

The following nanostructures were obtained, also inspired by the work of Bulfoni et al.^55^ NS-1 (see Fig. 1) were functionalized for the mesenchymal cells identification with anti-CD44 and anti-N-Cad Abs, and with Malachite Green as SERRS reporter. NS-2 were functionalized for epithelial cells identification with anti-EpCAM and anti-E-Cad Abs, and with Texas Red as SERRS reporter. NS-3 were functionalized, to recognize PBMCs, with anti-CD45 Ab and with Nile Blue as SERRS reporter. The UV–Vis-NIR extinction and Raman spectra of the three different nanostructures are reported in Fig. 1b. Compared to the pristine AuNPs, the NS-i show plasmon bands shifted to the near infrared region, characteristic of aggregated nanostructures^53^, providing a good resonance with the laser excitation at 632.8 nm used for the SERRS measurements. The different SERRS spectra of the three nanostructures (see Fig. 1c) allow easy multiplexing measurements, namely the simultaneous recording of the three types of SERRS signals in a single spectrum using only one laser excitation. Only a partial cell targeting activities of NS-1 and NS-2, were observed with standard flow cytometry (see Supplementary Fig. S2). This can derive from the fluorescence quenching induced by the presence of the plasmonic nanostructures, which reduces the reliability of these measurements. For this reason these data have not been used as reference points for the Random Forest analysis (see below).

### Tumor cells capture and targeting

**Figure 2:**
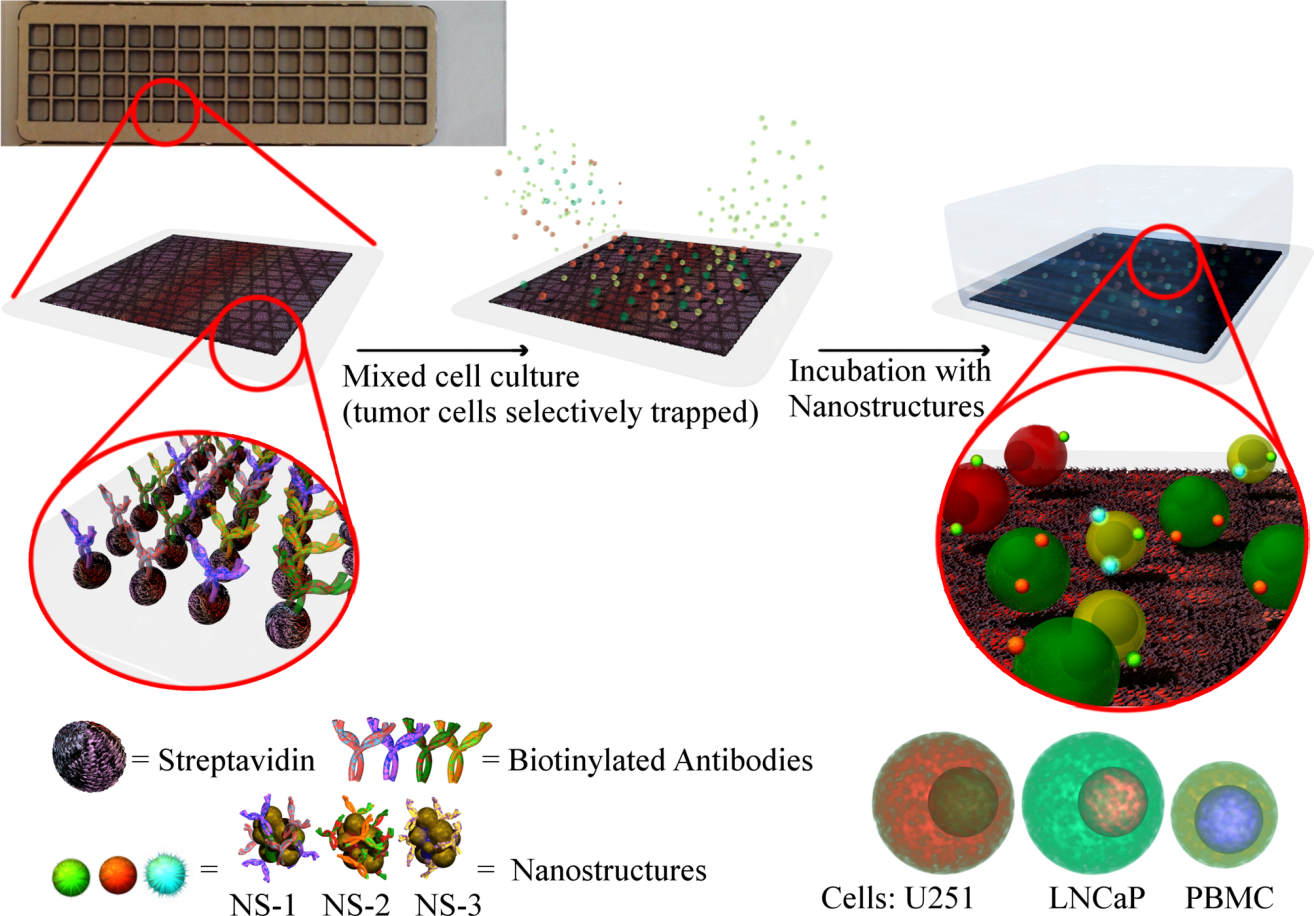
Schematic representation of the capture chamber, the tumor cells trapping and their incubation with a mixing of the three nanostructures (NS-1, NS-2 and NS-3), selectively recognizing the antigens of interest.

The protocol for the identification and enumeration of tumor cells in peripheral blood was applied to spiked samples in which a fixed number of tumor cells (about some tenths per ml, see below) were added to PBMCs (i.e. white blood cells, WBC) from healthy donors, separated by the whole blood by a standard density gradient centrifugation (see “Methods” below). This step, namely samples in which tumor cells and PBMCs are present after red blood cells of a whole blood sample are discarded, is the important step for their identification^13–15,55^. The spiked samples were obtained with a number of WBC corresponding to the amount present in 7.5 ml of blood (about 7 × 10^7^ PBMCs), which is considered a reference volume for such analysis^56^. Although this could be considered a limiting aspect of the preparation of the samples, it allowed focusing on the validation of the protocol for the identification and enumeration the tumor cells after their fixing on a slide (see below). The separation of the tumor cells in peripheral blood of a patient would follow the same protocol, and the tumor cells would be present together with PBMC. However, in this case an unknown number of tumor cells would be present, which is a problem for the validation of the protocol. The identification of low amount of tumor cells required a pre-analytical step in which the depletion of the large amount of WBC was obtained with an immunomagnetic sorting using anti-CD45-commercial magnetic beads (anti-human CD45 magnetic beads from Milteny) and following the manufacturer’s protocol. Depletion of the order of 99.99% was obtained, which, however, means that thousands of PBMCs continued to be present in the samples and that, therefore, their identification were always needed. After this step the tumor cells were trapped on a glass slide activated with avidin, and functionalized with the Abs also used for functionalizing NS-1 and NS-2. For the immobilization on a glass slide, Abs were first biotinylated to exploit the high biotin-avidin affinity and stability. The avidin activated glass slide was also divided into small chambers of 3.5 × 3.5 mm with an adhesive silicon mask to reduce the incubation chamber where the cells were trapped and incubated (see Fig. 2).

The tumor cells were captured within these chambers and fixed to improve their retention for the following steps, namely for the incubation with the NS-i and washings. The efficiency of the capture and fixing steps for LNCaP and U251 cells were determined by counting the cells on the slide after the final washing. The efficiency of this procedure was found to be 84 ± 7%.

Incubation of the cells was obtained with 0.5 nM solutions of NS-1, NS-2 and NS-3. The fixed cells were incubated with a mix of the three types of nanostructures for two hours at room temperature and than washed with PBS to remove all unbound nanostructures.

### SERRS spectra acquisition and tumor cells identification

SERRS spectra were acquired for each cell present in a chamber with a µ-Raman spectrophotometer using the 632.8 nm laser line. Spatial coordinates of all the cells present in a chamber were determined using an image recorded with the camera of the instrument and a 20 × objective. The real area of each cell was measured, using the image pixels, for the following analysis. Then the µ-Raman spectrophotometer collected the SERRS spectra for each individual cell.

The threshold for a positive targeting activity of the three nanostructures was obtained with the Pearson coefficients calculated by comparing the spectrum collected for each cell with the three spectra characteristic of the three SERRS reporters.

These data, together with the dimensions of the cells, were used for a Random Forest (RF) analysis. RF is a well known learning method for supervised classification problems and is an ensemble learner, that is, a method that generates classifiers and aggregates their results^49^. The RF analysis makes possible to assign to each cell a percentage of attribution to the three classes: PBMC, U251 (mesenchymal cell) or LNCaP (epithelial cell). The dominant attribution identifies the type of cell.

Reference samples, with only PBMCs, U251 or LNCaP cells were used for calibrating the RF analysis. Supplementary Figs. S3–S5 show examples of the spectra obtained for the different type of cells, after incubation with the three types of NS-i, mixed together, and washing to remove unbound nanostructures.

Pearson correlation coefficients for all the cells of the three reference nanostructures showed that NS-2 SERRS signals well identified LNCaP cells, as expected, whereas NS-1 and NS-3 SERRS signals were found both for U251 and PBMCs, although with different relative abundances. This result was not surprising considering that both monocyte and lymphocyte show a positive CD44 and CD45 expressions. A large amount of cells into the well was constituted by PBMCs since the complete depletion of PBMCs with the immuno-magnetic sorting was unworkable. For a better identification of the U251 cells, another information was used in the RF analysis to discard PBMCs. A simple analysis of the dimension of the cells showed that large part of the PBMCs had surface area smaller than 30 µm^2^, whereas for LNCap and U251 the area was larger than 30 µm^2^ with an upper limit of 180 µm^2^ (see Supplementary Fig. S6). Therefore, RF was instructed to consider only cells with surface larger than 30 and smaller than 180 µm^2^. Clearly, these limits can be modified although they are appropriate also for other tumor cells^57^.

Incubating three samples of a single type of cells with all the three nanostructures, the RF analysis allowed estimating the errors in the identification of the three types of cells (see Supplementary Table S1). One found that the best classified cells were the epithelial ones (LNCaP, 93% of cells identified), but also the mesenchymal ones (U251, 80% of cells identified) were satisfactorily identified.

A calibration of the RF analysis was then possible with a theoretical mixing of cells randomly chosen from the three samples with single type cells. In this case we knew the exact number of each type of cells present in the samples and they were compared with the RF output. RF gave a percentage of attribution to the identification of cells. 33% of percentage of attribution for the three types of cells, indicates uncertainty in the identification. The results obtained with the theoretical mixing showed (see Supplementary Fig. S7) that 60% of percentage of attribution identifies both LNCaP and U251, recovering the tumor cells of the theoretical mixing.

After the RF model calibration, two real spiked samples (Mix1 and Mix2) were constructed with virtually the same number of tumor cells. Nominally, they contained 24 ± 1 per ml of LNCaP and 29 ± 1 per ml of U251 mixed with a PBMCs amount similar to their number present in whole blood, namely about 10^7^ per ml. Uncertainties in the number of LNCaP and U251 cells were determined with ten replicas of the dilution needed for counting the cells.

The two samples (Mix1 and Mix2) were incubated, washed and the SERRS signals recorded for each cells as above explained. The data were then analyzed with the RF model.

**Figure 3:**
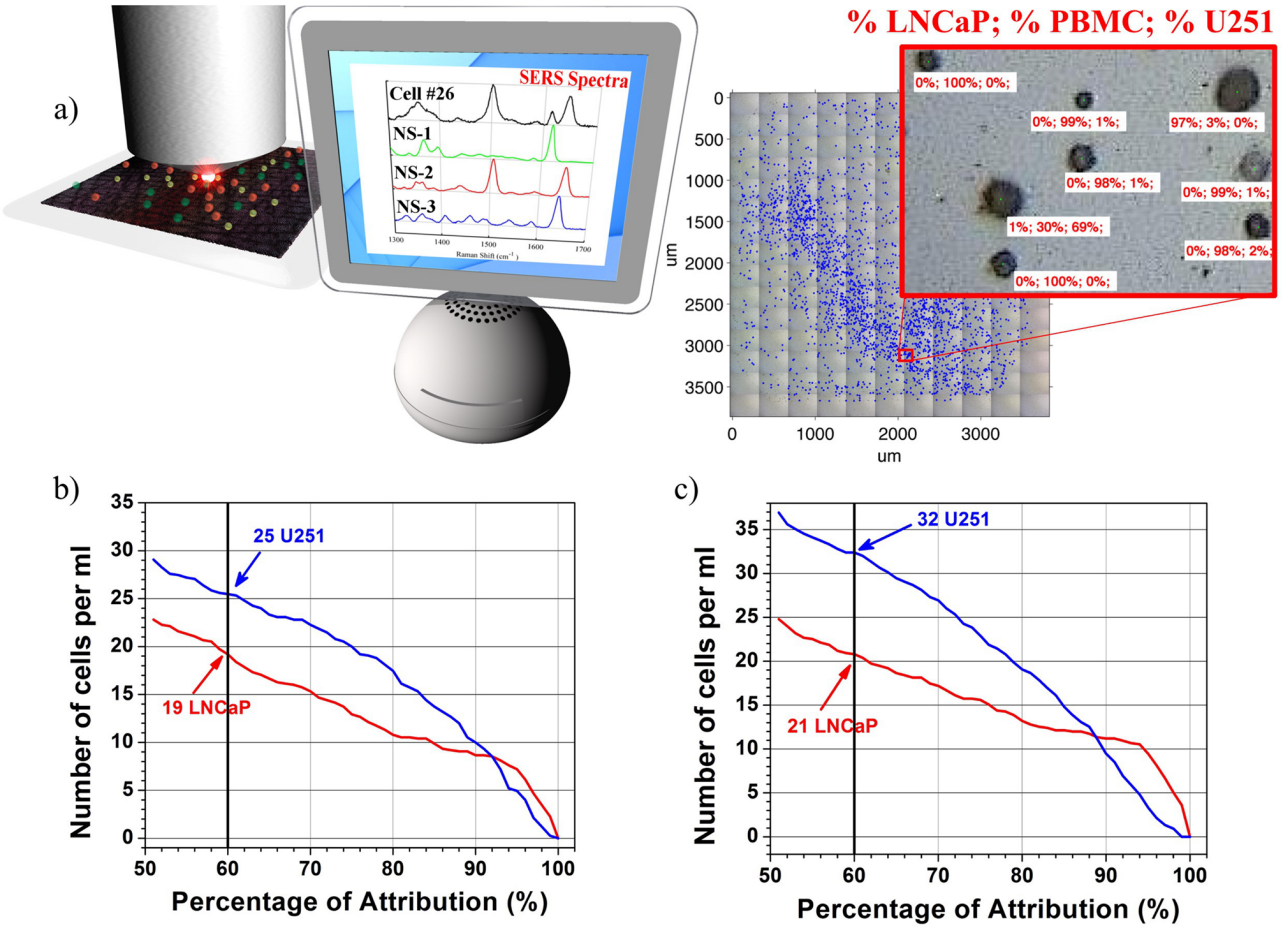
(**a**) Schematic representation of µ-Raman recording of the SERRS spectra of cells in a chamber slide. Thousands of cells were analyzed one-by-one and their SERRS spectra compared with reference ones. A Random Forest ensemble learning method was used to classify each cell as LNCaP, PBMC or U251. (**b**) and (**c**) The number of cells identified as a function of percentage of attribution given by RF for Mix1 and Mix 2 respectively.

In Fig. 3b,c the RF attribution result for Mix1 and Mix 2 are presented. Using 60% of percentage of attribution, previously determined with the theoretical mixing, RF recovered 19 ± 1 LNCaP and 25 ± 5 U251 in Mix 1, and 21 ± 1 LNCaP and 32 ± 6 U251 in Mix 2. The results are summarized in Table 1. One should recall that the recovered amounts are 84% of the initial population because of the capturing and fixing steps (see above). Considering the uncertainties reported above, the enumeration of LNCaP is very satisfactory, as well as that of U251 in Mix1, whereas an overestimation is found for the U251 cells in Mix2 (32 with respect to 24), which, however, is within the estimated errors for this type of cells.

**Table 1:** Number of spiked, fixed and identified tumor cells in Mix1 and Mix2.

Per ml	Mix1	Mix2
Spiked LNCaP	24 ± 1	24 ± 1
Spiked U251	29 ± 1	29 ± 1
Fixed LNCaP	20 ± 2	20 ± 2
Fixed U251	24 ± 2	24 ± 2
Identified LNCaP^a^	19 ± 1	21 ± 1
Identified U251^a^	25 ± 5	32 ± 6

^a^Based on a 60% percentage of attribution.

Considering a percentage of attribution higher than 60% (see Fig. 3b,c) a better cell identification can be obtained, although for a smaller number of cells. This can be useful for example for further analyses that can be made on single cells for studying other types of markers useful for a more complete understanding of a tumor stage.

## Conclusions

Tumor cells in peripheral blood are growing up as important elements for liquid biopsy due to their early cancer diagnostic potential. For this reason, accurate and specific assays are needed for their identification, characterization and enumeration. In the present approach surface enhanced resonance Raman scattering (SERRS) signals from plasmonic multivalent nanostructures and a Random Forest ensemble learning analysis allowed identifying the tumor cells of spiked samples. In particular, for their importance in a tumor progression analysis, mesenchymal and epithelial cells were considered and identified at the same time. The final identification is given as a percentage of attribution for each single cell. The results show that the present protocol could be efficient for circulating tumor cells (CTCs) identification and enumeration suggesting that it could be used for an early detection of a tumor, its recurrence and a precise and fast monitoring of a response to therapy.

## Methods

### Synthesis of gold nanoparticles (AuNPs) by laser ablation synthesis in solution (LASiS)

As previously reported^31^, AuNPs were synthesized using 9 ns pulses at 1064 nm of a Nd:YAG laser. The laser beam was focused with a lens (10 cm focal length) on a pure Au target at the bottom of a cell filled with 50 ml of 10^–5^ M NaCl aqueous solution. Fluence of 1 J/cm^2^ was used for obtaining nanoparticles with average diameters of 25 nm and a ζ-potential of about − 30 mV.

### Synthesis of thiolated antibodies

Anti-EpCAM (HEA-125 clone, Antibodies on-line), anti-ECadherin (67A4 clone, BioLegend), anti-NCadherin (8C11 clone, eBiosciene), anti-CD44 (IM7 clone, eBioscience) and anti-CD45 (2D1 clone, EXBIO) antibodies were used as targeting agents and functionalized with free thiol groups following the same experimental procedure already reported^31^. Briefly, 150 μl of 1 M solution of NaHCO_3_ were mixed with a solution of 2-iminothiolane hydrochloride (0.5 g/l) and 0.7 mg of one antibody. The reaction proceeded for two hours at room temperature and then overnight at 4 °C. The product was than purified by centrifugal ultrafiltration (5000 Da, Vivaspin 500 Sartorius) and washed for three times with a PBS (phosphate buffer saline, 50 mM PO_4_^3−^ and 150 mM NaCl at pH 7.4 added with EDTA 4 mM) solution. The functionalization grade was determined through a titration with 2,4-dithiodipyridine, resulting between 1 and 2 thiols per antibody.

### Synthesis of NS-1, NS-2 and NS-3

For NS-1, 8 ml of laser ablated AuNPs with 2.5 nM concentration, were aggregated by centrifugation at 25,000 RCF and re-dispersed in 1 ml of bidistilled water by sonication. 100 µl of a 50 µM hydro-alcoholic solution of thiolated Malachite Green^31^ (MG-SH) were added. The unbounded dye was removed by centrifugation. Then, 0.04 and 0.1 mg of thiolated anti-CD44 and anti-N-Cad were added. The reaction proceeded for 5 h at room temperature and overnight at 4 °C. The excess of antibodies was removed by centrifugation and the nanostructures re-dispersed in 1 ml of PBS. 100 µl of 0.5 g/l mPEG5000-SH (α-methoxy-ω-mercaptopolyethylenglycol, 5 kDa, Rapp Polymere) was added to complete the coverage of the gold surfaces. After 3 h of mixing at room temperature the nanostructures were purified by centrifugation. Finally, they were re-dispersed in 250 µl of PBS also adding 7 mg of BSA (Bovine Serum Albumin, Sigma Aldrich). The presence of antibodies on the surface of nanostructure was verified by the spectrophotometric analysis of supernatant after the reaction. The decrease of the characteristic absorption band at 280 nm of antibodies was used for determining the reaction yield.

The synthesis of NS-2 and NS-3 were performed following the same procedures, but changing both the SERRS reporter and the targeting Abs.

To construct NS-2 we used 100 µl of a 50 µM solution of thiolated Texas Red^31^ (TR-SH) as SERRS reporter, 0.05 mg of anti-EpCAM and 0.09 mg of anti-E-Cad, whereas, for NS-3, 100 µl of a 50 µM solution of thiolated Nile Blue^54^ (NB), and 0.14 mg of anti-CD45.

UV–Vis-NIR and SERRS spectra for the three nanostructures are reported in Fig. 1b,c. TEM images are reported in Supplementary Fig. S1.

### Cell cultures

LNCaP (human prostate cancer cells) and U251 (human malignant glioblastoma cells) were obtained from American Type Culture Collection (ATCC, Rockville, USA). The cells were maintained at 37 °C in a humidified atmosphere containing 5% CO2 and 90% of humidity. LNCaP cells were cultured in RPMI 1640 Medium supplemented with 2 mM L-Glutamine and 0.01 M HEPES. U251 cells were cultured in Dulbecco Modified Eagle’s Medium (DMEM) containing 3.7 g/l sodium bicarbonate, 4.5 g/l glucose and supplemented with Glutamine and HEPES. Moreover, all the media were supplemented with 10% heat-inactivated fetal bovine serum (FBS) (Invitrogen, NY, USA) and antibiotics (0.1 mg/ml streptomycin and 100 units/ml penicillin G, Sigma-Aldrich, St Louis, MO, USA). Before the experiments, cells were detached with PBS-EDTA to obtain a monocellular suspension. The cell dilutions applied to create spiked samples were counted ten times by a Neubauer chamber.

Blood was obtained from healthy donor and the study was approved by CRO (Centro di Riferimento Oncologico, Aviano, Italy) Ethical Committee (code: IRB-18-2011) and all participants gave their informed consent. Moreover, all experiments were performed in accordance with relevant guidelines and regulations. PBMCs were obtained from whole blood by a standard density gradient centrifugation according to the supplier guidelines (Ficoll-Paque, GE Healthcare)^58^.

### Incubation and trapping

To create the chamber for samples trapping/reading we used avidin-coated glass slide 75 × 25 mm (TRIDIA BA Slides, SurModics). To reduce the working chamber area to a 3.5 × 3.5 mm^2^ well we applied an adhesive multiwell gasket (FlexWell 64, GRACE BIO-LABS). After rehydration, the well surface was activated by incubation with a cocktail of biotinylated Abs (i.e. anti-EpCAM, anti-E-Cad, anti-N-Cad and anti-CD44 antigens) to trap the desired cell populations. We used around 0.25 µg of each Abs per chamber, with an incubation times of 30 min at room temperature. After three washing steps, cell samples (i.e. spiked samples, PBMCs only and tumor cells only) were incubated at room temperature for two hours. Then cells were fixed for one hour with PFA/Sucrose 4%, neutralized with Tris 10 mM pH = 8.0, washed with PBS and quenched with FBS. Staining was performed at room temperature for one hour using the targeting nanosystems preincubated in PBS and non-fat dry milk 5%. Finally, the chamber was washed with PBS and prepared for SERRS analysis.

### µ-Raman measurements

SERRS spectra were recorded with the µRaman instrument inVia Renishaw, equipped with a motorized stage and with excitation at 632.8 nm using 3 mW measured on sample plate. A 20 × LEICA objective and 1 s acquisition time were used. Since cell mixtures were fixed on the bottom of 3.5 × 3.5 mm^2^ wells, the entire surface was considered. Raman spectra were recorded for each single cell. Pattern recognition algorithms were used for cells identification in the chambers. The full colored image of a chamber was transformed into a binary image and a morphological operator detected specific cell patterns. Cells dimensions were also obtained by summing the image pixels of each identified cell. The centroid of each cell was recorded as xy coordinates and passed to the instrument for the SERRS measurements. All algorithms were developed in Matlab with graphical interfaces.

### Data analysis

For building the Random Forest (RF) classifier we used the RandomForest package of R^59,60^. We used the Pearson correlation coefficients to compare the recorded spectra of each cell with the SERRS signals of NS-1, NS-2 and NS-3. We considered only cells with diameter in the range 30–180 µm^2^. The pairwise Mann–Whitney test with the HOLM correction for multiplicity was applied in order to detect significant differences in terms of cell diameter between LNCaP, PBMC and U251. The RF classifier was built using 5000 trees, with the number of variables tried at each split, i.e. the mtry parameter, fixed at the default value, here equal to 3. The out of bag (OOB) error rate of the final RF model was 11.41% whereas the class error rates were: 6.6%, 11.3% and 20.1% for LNCaP, PBMC and U251, respectively. Adding interactions and squares or cubes of the features did not help improving the OOB error rate or the class error rates.

## Supplementary information

Supplementary Information.
