# Supplementary material for: SERRS multiplexing with multivalent nanostructures for the identification and enumeration of epithelial and mesenchymal cells
Source: Sci Rep. 2020 Sep 25;10:15805. doi: 10.1038/s41598-020-72911-w (PMC7519640; doi:10.1038/s41598-020-72911-w)
Supplement: Supplementary file 1 — Supplementary Information. [file 41598_2020_72911_MOESM1_ESM.pdf]

Supporting Information

SERRS Multiplexing with Multivalent Nanostructures for the Identification and Enumeration of Epithelial and Mesenchymal Cells

Lucio Litti, Andrea Colusso, Marcella Pinto, Erlis Ruli, Alessia Scarsi, Laura Ventura, Giuseppe Toffoli, Marco Colombatti, Giulio Fracasso, Moreno Meneghetti**

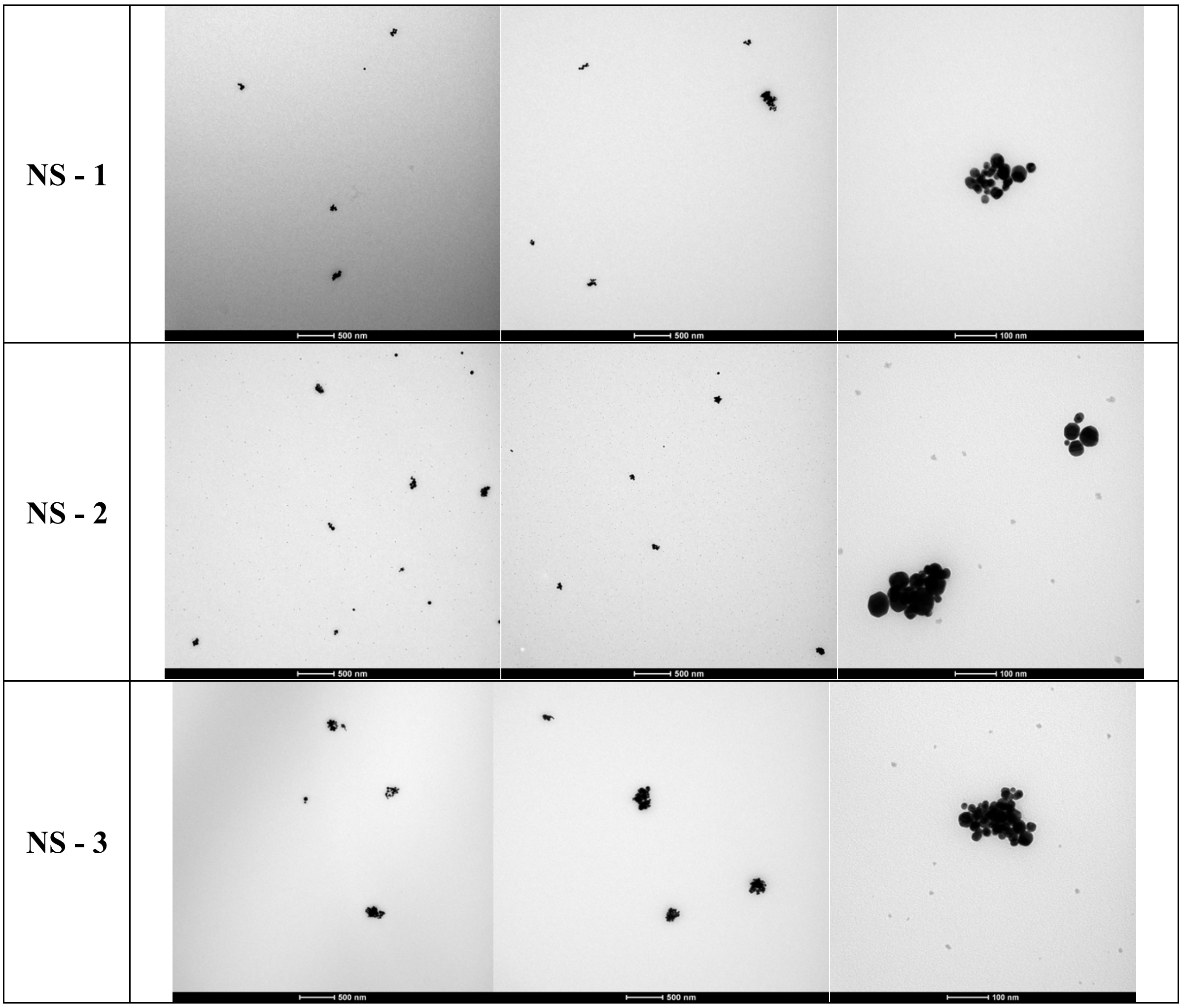

Figure S 1: TEM images for the three nanostructures NS-1, NS-2 and NS-3. In the first and second columns the bar below the images corresponds to 500 nm, whereas in the third it corresponds to 100 nm. We found average dimensions within 100 - 200 nm for all the nanostructures.

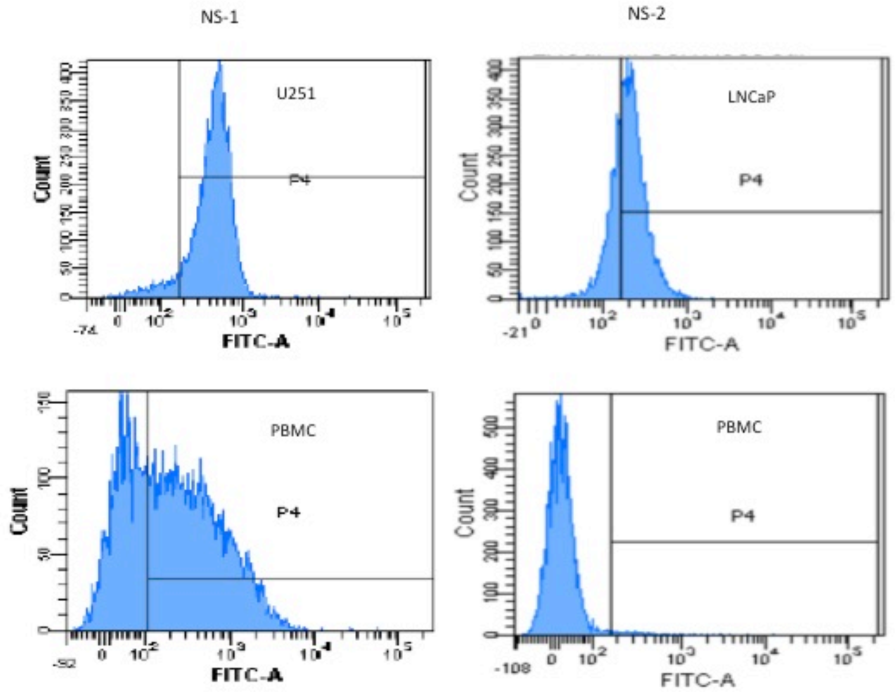

Figure S 2: Activity of NS-1 and NS-2 for U251, LNCaP respectively and of both for PBMC with a FACS analysis. The data give only a qualitative indication of the activity because of the fluorescence quenching activity of the plasmonic nanostructures.

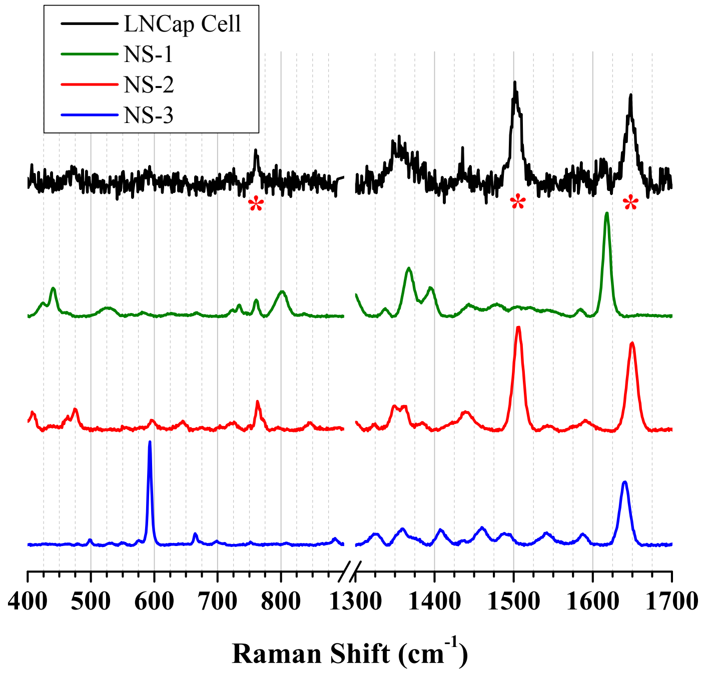

Figure S 3: SERRS spectra of an LNCaP cell after incubation with all three types of nanostructures and washing. Below are the reference SERRS spectra of NS-1, 2 and 3. In the cell spectrum asterisks indicate the relevant features of NS-2.

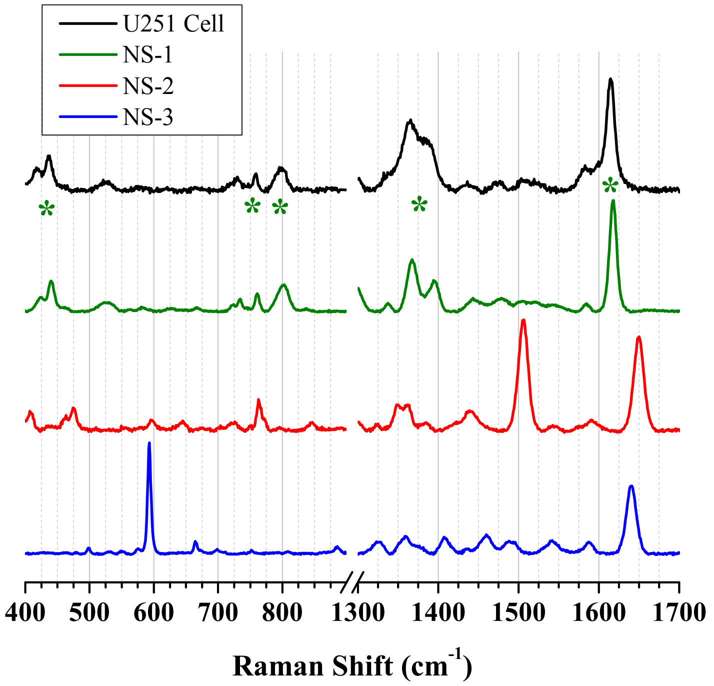

Figure S 4: SERRS spectra of a U251 cell after incubation with all three types of nanostructures and washing. Below are the reference SERRS spectra of NS-1, 2 and 3. In the cell spectrum asterisks indicate the relevant features of NS-1.

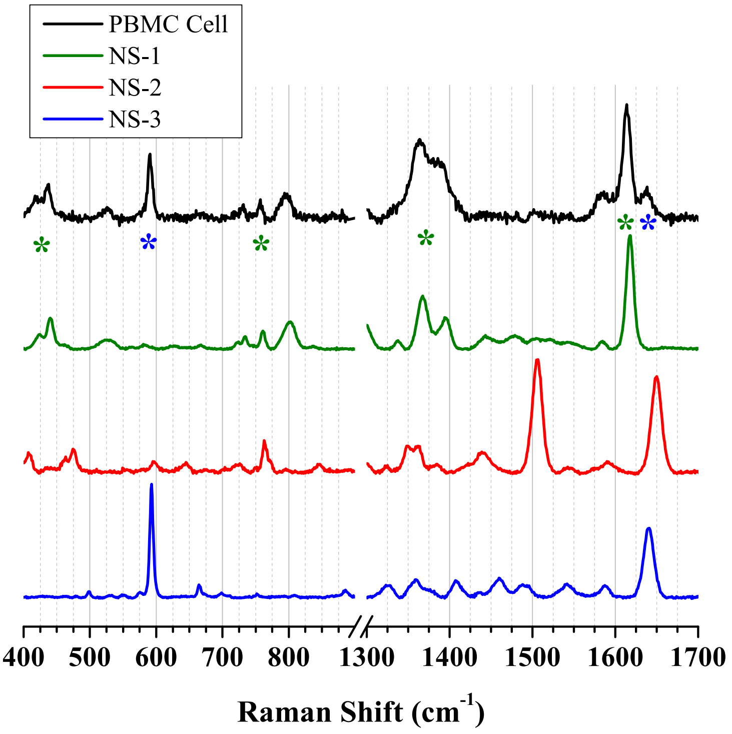

Figure S5 SERRS spectra of a PBMC after incubation with all three types of nanostructures and washing. Below are the reference SERRS spectra of NS-1, 2 and 3. In the cell spectrum asterisks indicate the relevant features of NS-1 and NS-3.

	Classified as LNCaP	Classified as PBMC	Classified as U251	classification error
LNCap	142	6	4	6.6 %
PBMCs	34	798	66	11.1 %
U251	13	19	127	20.1 %

Table S 1: RF confusion matrix

Table S1 gives the estimated classification error rate for each kind of cell contained into the theoretical mixing, while the entire model has a percentage error of less than 12%.

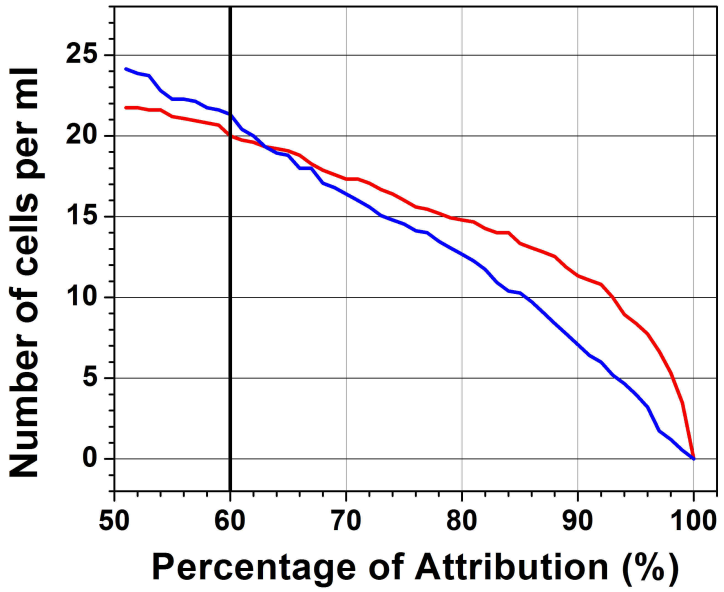

Figure S6. Amount of cells found against percentage of attribution. Increasing the percentage of attribution the correct identification of a cell is increased, but lower number of cells are identified. 60% percentage attribution can be considered the best value for the identification.
